# Research into Specific Mechanical Properties of Composites Produced by 3D-Printing Additive Continuous-Fiber Fabrication Technology

**DOI:** 10.3390/ma16041459

**Published:** 2023-02-09

**Authors:** Peter Pokorný, Daynier Rolando Delgado Sobrino, Štefan Václav, Jana Petru, Rafał Gołębski

**Affiliations:** 1 Institute of Production Technologies, Faculty of Materials Science and Technology in Trnava, Slovak University of Technology in Bratislava, Ulica Jána Bottu 25, 917 24 Trnava, Slovakia; 2 Faculty of Mechanical Engineering, VŠB—Technical University of Ostrava, 70800 Ostrava, Czech Republic; 3 Faculty of Mechanical Engineering and Computer Science, Department of Technology and Automation, Częstochowa University of Technology, 42-201 Czestochowa, Poland

**Keywords:** composites, CFF technology, mechanical properties, computer tomography, 3D printing

## Abstract

This paper introduces novel research into specific mechanical properties of composites produced by 3D printing using Continuous-Fiber Fabrication (CFF). Nylon (Onyx) was used as the composite base material, while carbon constituted the reinforcement element. The carbon fiber embedment was varied in selected components taking values of 0°, 45°, 90°, and 135° for parts undergoing tensile testing, while one specific part type was produced combining all angles. Carbon-fiber-free components with 100% and 37% fillings were also produced for comparison purposes. Parts undergoing the Charpy impact test had the fibers deposited at angles of 0° and 90°, while one part type was also produced combining the four angles mentioned before. Carbon-fiber-free parts with 100% and 37% fillings were also produced for comparison purposes as with the first part. The Markforged MARK TWO 3D printer was used for printing the parts. These were subsequently scanned in the METROTOM 1500 computed tomography and submitted to the tensile and impact tests. The results showed that adding carbon fiber to the base material increased the volume of defects in the samples as a result of the porosity increase. Although the tensile testing manifested an overall increase in tensile strength Rm of up to 12 times compared to the sample without reinforcement, it was proven that an improper fiber orientation significantly diminished the strength and that combining the four selected angles did not lead to the highest strength values. Finally, the impact tests also showed that fiber-reinforced parts implied up to 2.7 times more work to fracture, and that an improved fiber orientation also led to strength reduction.

## 1. Introduction

Usually, a material that consists of two or more components with different properties and a clear interface or limit between the individual components is referred to as a composite material. It is well known that these composites have different properties arising from the same components residing in them. Composite materials are used in various industries such as construction, shipbuilding, automotive, and aerospace. In the engineering industry, they are commonly used in the form of measuring and welding elements, in different grippers’ jaws, as components of diverse fixtures and clamping devices for CNC machining, as functional end parts or tools with high mechanical strength, and in the prototyping of parts as replacements for those obtained in more conventional machining processes [1].

In this regard, it has been documented in the states of the art and practice that the 3D printing production process has a great influence on the properties of composites. Similarly, it has also been discussed that the choice of materials that make it possible to meet given performance requirements in a final part or component also implies a series of trade-off decisions and/or compromises between several factors that may end in having a pronounced impact on the obtained composite. In this sense, there has been much relevant research; see, for example, [2], where the mechanical properties of a given composite in a combination of polyamide and continuous carbon fibers have been properly boarded. In their paper, these authors created a hierarchical structure of criteria enabling and facilitating the selection of the proper material while also taking into account physical, economic, environmental, and social impacts. They applied the results of the analyses to the construction of a high-speed train, and in consequence, their research confirmed that such an approach to composite material selection could be an effective tool for designers in general. Similarly, the study presented in [3] examined in detail 3D-printed composite parts by analyzing and comparing the mechanical properties of different composites as well as various production techniques used for their production. This work also provided a detailed review of the scientific literature including current trends and areas of application of composite materials. On the other hand, the authors in [4] studied a composite made of nylon, which was reinforced with fiberglass fiber. They concluded that such composites could be used for complex parts in industry, while also reaching with their analysis a dimensional accuracy of ±0.15 mm and a surface roughness of Ra = 5.3 µm. This research also recommended that in order to shorten the production process, the density of the filling was to be reduced. In a study presented in [5], the authors also focused on the development of fiber-reinforced materials, while mentioning some shortcomings of composite materials in relation to their mechanical properties, and also pointing out clear gaps in the current states of the art and practice when it came to predicting the performance of composites.

On the other hand, it is generally well known that composite fibers can be oriented in different directions in a 3D printer. There are usually three different approaches when laying down the fiber into the plastic matrix (see [6,7,8]), i.e., (1) depositing the fiber before the printing process, (2) incorporating the feeder into the same printing head, and (3) laying it down directly on the plastic matrix. The authors in [9] built their own 3D printing device using an experimental print head. This printer was capable of extruding carbon-fiber-reinforced composites, and during this study, they used an ABS matrix reinforced with 10% of continuous carbon fiber. The results of this research also showed that the flexural and tensile strengths of the composite presented higher values when compared to those obtained in a classical injection molding process, where the fiber content is the same. In particular, the flexural strength values increased to a total 127 MPa, while the tensile strength values increased to 147 MPa, leading these authors to logically conclude that the 3D printing of fiber-reinforced components had a great potential in the production of composite parts in practice. In the case of the research presented in [10], several samples were printed using the free-hanging printing method. The printed samples had diverse shapes, i.e., quadrilateral, pyramidal, circular grid, and others. The study compared printing accuracy; examined print morphology, stresses and deformations, relative density, and fiber volume fraction; compared these values with existing well-documented lattice structures. The authors concluded that by placing a continuous fiber in a polymer matrix, it was possible to significantly improve the functional properties of the parts, while also stating that other advantages lied in lower costs for the production of parts from composite materials and in the possibility of optimizing the topology of these.

Similarly, the study in [11] compared two types of fibers used for the additive production of parts; it presented price differences and uses of both carbon and glass fibers, while it also listed areas of industry where both of these fibers were commonly used because of low-weight and high-strength requirements (aerospace, aviation, and sport industries). The parts presented in the paper were produced using a unidirectional placement of the fibers at an angle of 0°, while the authors also analyzed the microstructure, density, and porosity of such parts, and performed tensile and bending tests as well as a quasi-static indentation force test. At the end of this study, the authors identified the advantages, possibilities, but also limitations of reinforcing plastic components with carbon and glass fibers, and more specifically, they also came to the conclusion that it was possible to produce high-performance parts with these composites due to, mainly, their high tensile strength if compared to unreinforced parts. In addition, the research presented by [12] investigated parts in which 70% of their volume was a polymer and the remaining 30% contained a continuous natural fiber. In this case, parts investigated in the paper were printed with XZ and YZ orientations with respect to the 3D printer platform. At the end of the research, the authors concluded that the porosity of the parts was around 4%, which, if compared to glass- and carbon-fiber-reinforced parts (approximately 15%), was considered as a relatively small value.

The study presented in [13] dealt with the research of carbon- and glass-fiber-reinforced ABS parts. In the production of experimental parts, the authors used an approach that lied in depositing the reinforcing fiber after the nozzle. Tensile strength tests and Young’s modulus were performed on the parts, leading to an increase of 17% and 21%, respectively, with a simple fiber volume in the parts of 0.6%. On the other hand, the authors in [14] used in their experiment a nylon matrix (Onyx) reinforced with Kevlar fiber. In this study, they proposed and investigated several Kevlar fiber deposition strategies, i.e., triangular, rectangular, and hexagonal types of fills. The samples were made on a Markforged Mark Two 3D printer, while the Markforged Eiger software (https://markforged.com/software, accessed on 7 June 2022)was used to prepare the assembly and simulate fiber placement. After extrusion, the samples underwent the Charpy impact test and a ballistic test using a gas weapon. In the Charpy test, the sample was oriented in two directions, i.e., Z and XY, which were determined by the position V of the groove on the sample. The authors found that the sample oriented in the Z direction had better properties than the sample in XY. They also concluded in the study that the reinforcement had no effect if the fibers were placed parallel to the V-groove, while on the other hand, the best results were obtained with samples reinforced with Kevlar fibers using a rectangular type of filling. Closely related to the previous research, the study in [15] also presented research into composite materials with a carbon-fiber-reinforced Onyx plastic matrix. The produced samples were tested by tensile and bending tests, and the results showed delamination failure as the main cause of component failure in the tensile tests. These findings led the authors to identify a problem in the bonds between subsequent layers and adjacent fiber layers, while they also calculated tensile and flexural strengths of about 560 MPa and 271 MPa respectively.

In the works presented by [16,17], the authors identified and presented a key step forward in the creation of composite materials that are reinforced with carbon fibers, by introducing patented technologies for the production of such composites. They divided these patents in two directions, i.e., patents for new production methods and patents for new structures of composite materials. During their work, these authors also reported an improvement in the mechanical properties when using continuous fibers over short fiber in the production of the composites. At the same time, they also concluded that the strength and stiffness of continuous-fiber-reinforced composites (produced by 3D printing) were still lower than the ones found in conventionally produced components, which is known to be a disadvantage of these materials, limiting their use in industry where tough requirements are placed on their mechanical properties. This study also identified the reason for the low strength, which lied in the same creation of cavities during 3D printing; in other words, they concluded that if fibers are incorporated into the matrix, the porosity increases, making the interfacial bond between the polymer and the fiber weak as well. Similarly, the authors in [18] also investigated the mechanical properties of fiber composite materials produced by additive technology. They compared them with composites made by conventional pressing. In this study, an ABS carbon-fiber-reinforced matrix was used to make the samples. The production of samples was realized on three different 3D printers, and the results showed different mechanical properties in the extruded parts as a result and/or consequence of the specific printer used. In addition, the manufactured parts had differently oriented fibers in the ABS matrix, and as it is well known that the mechanical properties of the parts also depend on the same direction of fiber placement, it was also possible for the authors to conclude that when comparing their 3D-printed extruded parts to parts made by conventional pressing, the extruded ones showed worse properties in each case.

In the same area of research, the paper in [19] had as a goal to investigate the mechanical properties of carbon-fiber-reinforced polymer matrix parts. Tensile and bending tests were performed, while the fiber volume in the matrix was varied. One type of part was reinforced with fibers of different orientations. The fiber volume variation in the matrix was performed by changing the number of layers of fibers. Next, the authors extruded a part with a carbon fiber Onyx matrix. Subsequently, a pressure of 50 bar was applied to the part at a constant temperature (130 °C) for 1 h, leaving the orientation of the fibers along the sample to bear the greatest load. It was found that by applying a pressure and temperature for 1 h, the structure between the layers improved as a result of lower porosity. The authors also found a significant increase in tensile strength of up to 768 MPa and modulus of elasticity of up to 80 GPa compared to non-pressed samples. Other research (see [20]) also made parts by 3D printing, investigating their resulting mechanical properties. In this case, the base material was also Onyx, which was reinforced with carbon fibers. The properties investigated were flexural strength and flexural modulus. Both of these works discussed above allowed the authors of this paper to reinforce their opinion once more on the fact that the distribution of fibers in the matrix is very important because it may lead to an uneven distribution of stresses in the part. For example, if the direction of fiber deposition is conceived in the direction of the force, the structure is significantly strengthened. Knowledge like this allows designers to position and orient the fibers into precisely defined areas of the parts in order to achieve the best possible properties in the end product. 

An extensive study of composite materials was also carried out in paper presented in [21]. The authors shared key knowledge about production techniques used for the production of composite materials. The last chapter of this publication is dedicated to the application areas in which composite materials are used. The researchers managed to improve the functional properties of the materials, and as mentioned before, these composite materials were identified to be of great value and use in industries such as aerospace, chemical, sports, and automotive. 

As for the classification of composite materials based on the individual components of the composite, the works in [22,23,24] offer a good approach to this. The authors based their classification with regard to (1) the size (nanocomposites), (2) the reinforcement (fiber, particles, shaping), (3) the matrix material (polymer, ceramics, metal), and (4) biocomposites. Composite materials with different matrix materials (epoxy resin, polyester, Cu, PLA, etc.) are clearly presented and discussed in [25,26,27,28,29,30,31]. In these publications, the matrices were reinforced with various materials (sheep wool fibers, TiO2 particles, jute fibers, etc.). In addition, such composites proved to improve properties such as impact resistance, heat resistance, tensile and flexural strength, and wear resistance. In this last classification of composites, it is also important to highlight the very matured and complex research presented by [32] in the field of high performance and/or more novel thermosetting resins such as the cyanate ester/benzoxazine one. These authors clearly proved the effect and relevance of silane in terms of treating the carbon fibers for the creation of composites using the mentioned resins. Likewise, it is also important to mention the research carried out by [33,34]; in the first one, the authors clearly investigated structural, morphological, mechanical, and thermal properties of newly designed polymeric materials using, again, high-performance hybrid fibers to reinforce the polybenzoxazine resins. In this paper, the authors clearly demonstrated and drew key conclusions on the progressive enhancement in the thermal and mechanical properties in the hybrid composites as a result of their treated fibers. As for the case of [34], the authors presented interesting research that boarded hyperbranched-polymer-coated carbon-fiber-reinforced DCBA/BA-a composites, while conducting detailed microscopic analysis (SEM) of their structural, morphological, and mechanical properties, allowing them to verify positive improvements in terms of modulus, bending, and impact strength in the analyzed specimens as a result of a better dispersion and adhesion of the coated carbon fibers within the resin matrix.

In general terms, it is possible to state that the improved properties of composites usually make them useful materials in various application areas such as mortars and plasters in tribological applications, in heat exchangers, or in the construction industry, where several improvements have been made in recent years; see, for example, [35], where the authors implemented short alkali-resistant glass fibers into fine-grained concrete, leading to an improvement in several mechanical properties, or the work presented in [36] where the authors focused on using agricultural waste in the creation of eco-friendly cementitious composite materials. Of course, the success of composites greatly depends on the individual production techniques used in their production, i.e., cold pressing, injection molding, and pressing. However, nowadays, there is no doubt that a key current trend in the production of parts relies more on additive technologies, which are mainly used for rapid prototyping of complex shapes, and where polymers or fiber-reinforced polymers are used as the base material. At present, metals are also used for the additive production of parts; for example, the work in [37] properly investigated some of their properties. However, it is still safe to say that additive technologies remain a tool primarily used with polymers, although there are even authors who have stated (see [38,39]) that fiber-reinforced polymeric composite materials (of various types and compositions) are in competition with some metals, e.g., aluminum itself.

As covered up until this point in this paper, it is clear that some significant research has been already performed and implemented in the area of fiber-reinforced polymeric composite materials. However, the authors of this paper have found that there are still gaps and poor coverage in the states of the art and practice in terms of analyzing how the selection of the proper fiber deposition orientation angles influences the resulting composite, especially if compared to other unreinforced materials. In addition, the consulted literature has also shown that there are only few studies that have used non-destructive tests in the investigation of composites and, hence, the importance and scientific and practical soundness in the use of computer tomography in combination with classic tests as they are performed are presented in this research. 

In this sense, the present paper focuses on the analysis of selected mechanical properties of parts produced by 3D continuous-fiber fabrication technology, which, in the case of polymers, is usually referred to in the scientific literature as CFRPs (Carbon-Fiber-Reinforced Polymers). The basic material for the research of the mechanical properties is nylon filled with micro-carbon fibers (Onyx); this material is the base for the Markforged composite parts and is reinforced with continuous carbon fibers. In the individual parts undergoing tensile tests, the positioning or carbon fiber orientation is changed at different parts of the process. In the specific case of one single sample, the fiber placement is performed in a combination of angles with values of 0°, 45°, 90°, and 135°. In the case of other samples, fibers are placed at angles of 0° and 90°. For comparison purposes, carbon-fiber-free parts with 100% and 37% fillings are also produced. For the Charpy impact test, parts with a fiber orientation of 0° and 90° are produced as well. As with the tensile test part, another sample is made in a combination of all the same four angles mentioned before. In order to compare the reinforced and unreinforced parts, carbon-fiber-free parts with 100% filling and with 37% filling are also produced for this case. The production of parts takes place on a Markforged (Watertown, MA, USA) MARK TWO printer. The components are subsequently scanned using a highly precise Zeiss (Oberkochen, Germany) METROTOM 1500 computed tomography system. The non-destructive tomography system allows for observing in detail the distribution of fibers in the matrix of the base material, previous to the realization of tensile and impact tests. This will allow the authors to better understand the whole experimentation process afterward and draw relevant conclusions about it.

Taking into consideration all of what has been mentioned in the above elements, the main aim of the research is to analyze how a controlled orientation of the reinforcing element during the design and production process may influence and possibly lead to the improvement of the mechanical properties of the resulting composites. This is to be performed and/or complemented by properly comparing these properties with those of unreinforced materials while making use what will allow the author to draw further conclusions from the study. This is a research space that has not been sufficiently investigated in the states of the art and practice, especially if taken into account that this paper aims to use either classical destructive or non-destructive tests for these purposes at the same time. All these elements mentioned above enhance and constitute the practical and scientific novelty of this research.

## 2. Materials and Methods

As mentioned in the previous section, the material to be used as matrix was Onyx, which is a nylon that contains micro-carbon fibers in its structure, and that is characterized by high strength, toughness, and chemical resistance [40]. The flexural strength is stated to be 71 MPa, which happens to be 1.4 times higher than that of ABS. In addition, Onyx is a material that can be even further fiber-reinforced. In this study, a continuous carbon fiber was used as the reinforcing material; when Onyx is reinforced with this fiber, it has been proved to achieve up to six times higher strength and eight times higher stiffness, and, thus, this composite has found application in the production of parts that are to replace aluminum in many cases, which enhances its relevance and its use potential for many industrial sectors [41]. 

For the purposes of programming the test samples, the Eiger software was used within this research. This software allows for setting composite printing parameters and then generating print paths. On the other hand, for the purposes of producing the parts, a Markforged Mark Two 3D printer pertaining to the company ADMASYS SK s.r.o. (Šaľa, Slovakia) was used. 

As also mentioned before, in order to scan the parts and observe the deposition of fibers in the matrix, the Zeiss METROTOM 1500 computed tomography system was used. As it is well known and has been stated in [42], computed tomography is one of the best non-destructive methods for evaluating the internal structure of materials. In addition, at present, this measuring method is increasingly used and is compared with coordinate measuring machines and their techniques; for example, the authors in [43,44,45] clearly pointed out the benefits of applying computed tomography measurements over other methods. 

On the other hand, it is also well known that mechanical tests are used to determine the mechanical properties of materials, in which a test specimen made is exposed to the action of mechanical forces. The force applied to the test specimen may be static, and in consequence, it is possible to break any material by tensile stress under extreme load conditions.

In this context, the Charpy impact test was used to evaluate the amount of energy absorbed by a material during fracture. The test was performed on a pendulum hammer and the energy absorbed by the parts was, in consequence, determined (see [46]).

## 3. Results and Discussion

**Figure 1:**
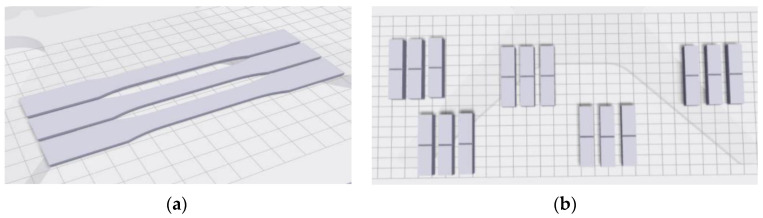
Allocation of test sample models on the 3D printer’s workspace in the environment of the Eiger software: (**a**) tensile test samples; (**b**) Charpy impact test samples.

The modeling of the experimental samples was the first step in experimental research; these samples had the same shape as the tensile test ones. Similarly, samples for the Charpy impact test were also modeled. Subsequently, these models were allocated on the 3D printer’s workspace in the environment of the Eiger software (see Figure 1).

The main monitored parameter was the distribution and/or orientation of the carbon fibers in the base material Onyx; this is something that can be set in the same Eiger software. The experimentation continued with the creation of four different types of composite samples. The first type created was a sample where the orientation of the carbon fibers changed after each layer, which resulted in a sample combining the fiber orientation at angles of 0°, 45°, 90°, and 135° in a single specimen (Type A). In particular, the first 4 layers were only of Onyx, the next 12 layers were Onyx + carbon fiber with the 5th layer having a 0° orientation, the 6th layer 45°, the 7th layer 90°, the 8th layer 135°, the 9th layer 0°, the 10th layer 45°, the 11th layer 90°, the 12th layer 135°, the 13th layer 0°, the 14th layer 45°, the 15th layer 90°, and the 16th layer having an orientation of 135°. The last 4 layers had no carbon fiber. Thus, in total, the whole set of angles (0°, 45°, 90°, 135°) was used 3 times. The last 4 layers were also made of only Onyx as well. This is an approach that has not been properly explored in the consulted literature and, given the many positives that seem to be involved in it, the authors wanted to fully investigate this course of action.

**Figure 2:**
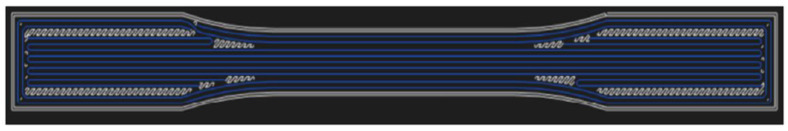
Carbon fiber oriented at an angle of 0°—sample for tensile test.

**Figure 3:**
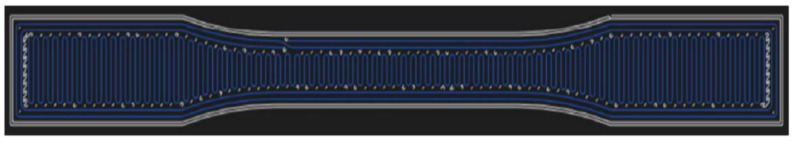
Carbon fiber oriented at an angle of 90°—sample for tensile test.

Next, a sample with a fiber orientation at an angle of 0° (Type B) was made (see Figure 2). On the other hand, the next sample (Type C) had the fibers oriented at an angle of 90° (see Figure 3). In order to compare the samples, carbon-fiber-free samples were also made out of Onyx material only. These samples were made as: Full Core Solid Fill 100% (Type D) and Infill Triangle Network 37% (Type E).

**Figure 4:**
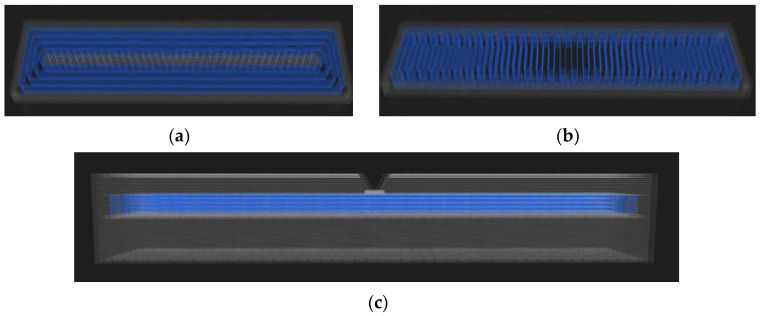
Carbon fiber orientation: Samples for the Charpy impact test: (**a**) fiber orientation at an angle of 0°; (**b**) fiber orientation at an angle of 90°; (**c**) side view of the sample and the beginning of the carbon fibers under the notch.

In Figure 4, it is possible to see a summarized take on the Charpy impact test. In this regard, it is convenient to specify that the parts for the Charpy test were made of 80 layers. The carbon fiber content was 20%, so 16 layers contained carbon fiber. Carbon fibers began to be deposited only in the first layer under the notch. The notches were printed. 

### 3.1. Scanning of the Printed Samples

**Figure 5:**
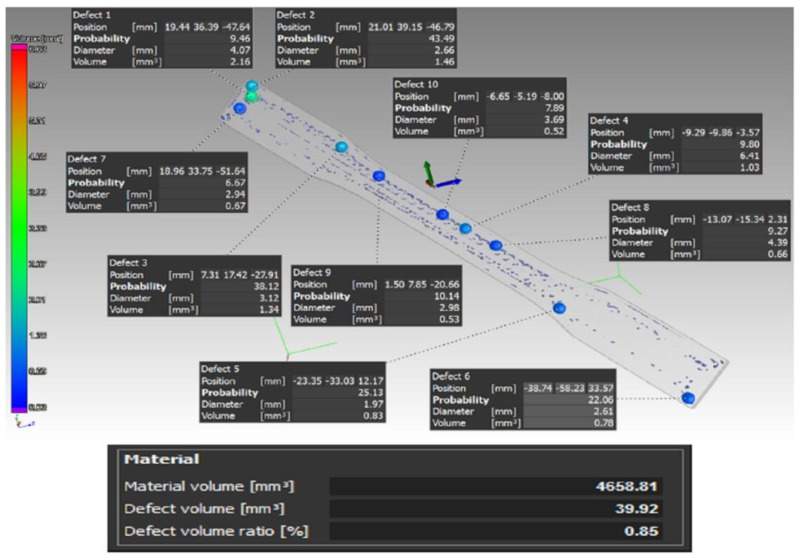
Distribution of the defects and their volume in the case of the type A sample.

After printing the samples that were to undergo the tensile and the impact tests, these proceeded to be scanned first. In this case, the VGStudio MAX 3.0 software was used to evaluate the results of the scanning process. A total of three pieces were made from each type of sample. Figure 5 showcases a scan example of a type A sample. 

The percentage of defects in the individual samples of type A was A1—0.85%, A2—0.47%, and A3—0.71%; these percentages relate to the whole sample volume.

**Figure 6:**
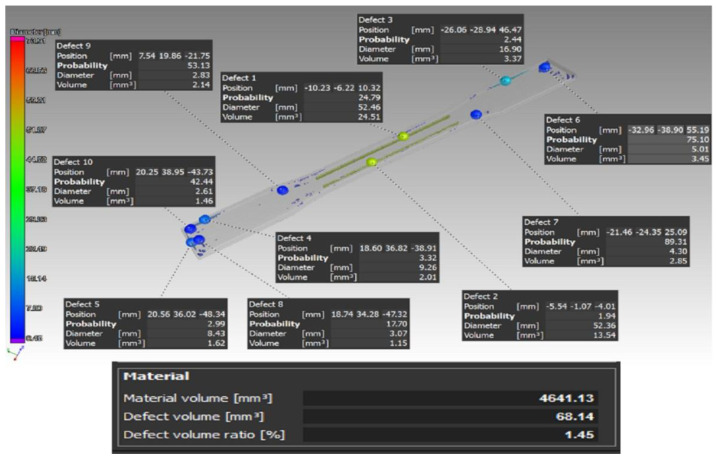
Distribution of the defects and their volume in the case of the type B sample.

Figure 6 shows a scan of the type B sample.

Similarly, the percentage of defects of the sample volume in individual samples of type B was: B1—1.45%, B2—1.22%, and B3—1.05%. Here, a clearly visible defect is shown in yellow on both sides of the sample. This phenomenon was caused by a large gap between the carbon fibers in the program generated by the Eiger software. This gap was present because the diameter of the inserted carbon fiber was 0.125 mm and because it was not possible to insert the fibers evenly in the sample, due to its shape and size. In this regard, the Eiger software calculated and determined the gap with respect to the carbon fiber diameter (the fiber could not be inserted in this gap), and in consequence, this appeared to be a defect in the scans, when in fact, it was not.

**Figure 7:**
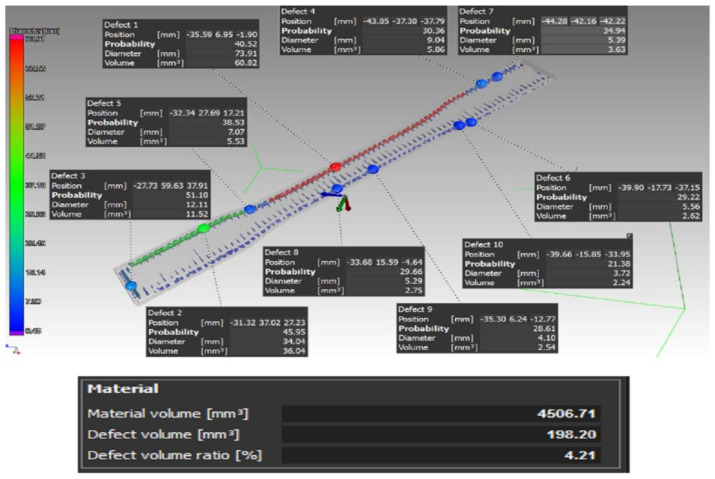
Distribution of the defects and their volume in the case of the type C sample.

Figure 7 presents a tomographic scan of a type C sample.

This type of carbon fiber storage insertion caused the largest number of defects compared to the previous types of carbon fiber deposition. The percentage of defects of the sample volume in individual samples was: C1—4.21%, C2—2.69%, and C3—2.67%. The defects in this case were distributed around the perimeter of the sample and, at the point of occurrence of defects, the carbon fiber generally bended by a total of 90°. It was precisely in such places where it was not possible to fill the space with the Onyx base material, which led to the appearance of such defects.

**Figure 8:**
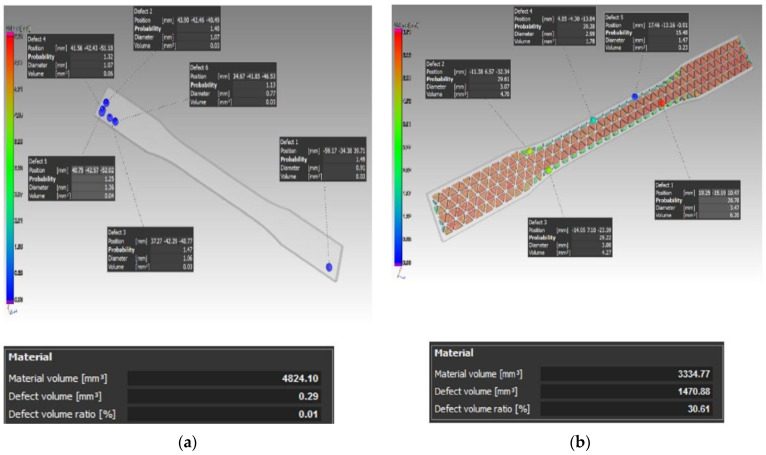
A general view on the distribution of the defects and their volume in the case of the samples without carbon fibers: (**a**) type D sample; (**b**) type E sample.

Figure 8 shows scans of the type D and type E samples where it is possible to see the distribution of defects. These were carbon-fiber-free comparative samples.

Similarly, as for the case of the percentage of defects of the sample volume in individual samples of type D, the results were: D1—0.01%, D2—0.05%, and D3—0.06%, while in the case of sample type E, the percentage of defects in individual samples was: E1—30.61%, E2—30.56%, and E3—30.53%.

**Figure 9:**
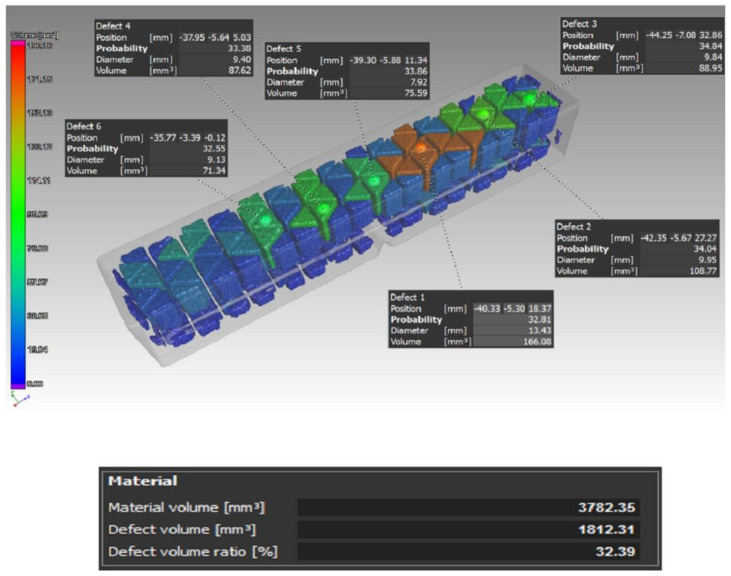
Distribution of the defects and their volume in the case of the type B sample for the purposes of the bending impact test.

In a similar way, the samples for undergoing the Charpy impact test were also scanned. All of the samples were made by the triangular infill strategy with the exception of the type D sample. For this reason, the detected defect values in volume were also high. Due to the use of this type of strategy, it was not possible to evaluate the effect of fiber deposition on the volume of defects in the impact test specimens. Figure 9 shows a type B sample scan before the sample was submitted to the impact test.

**Figure 10:**
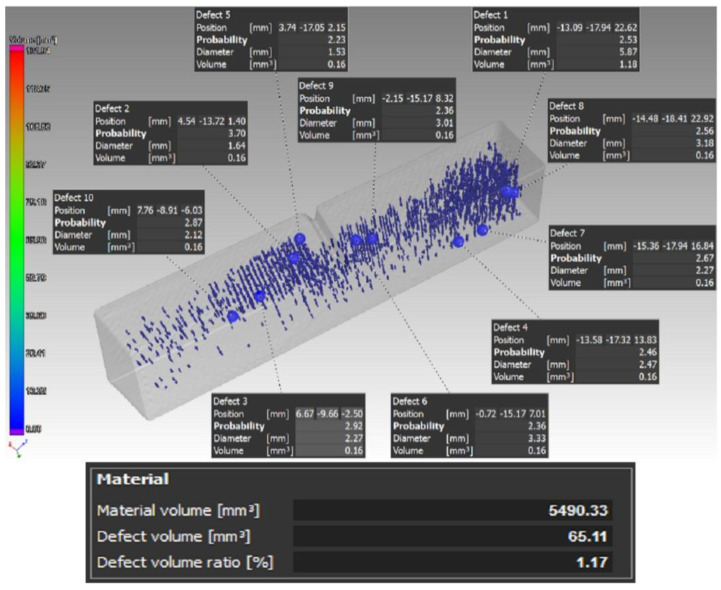
Distribution of the defects and their volume in the case of the type D sample for the purposes of the bending impact test.

An example of the porosity in the type D samples can be seen in Figure 10. As mentioned before, in this case, carbon fibers were not used as reinforcement for the Onyx base matrix, and the whole sample was extruded with a solid core. For each of the individual samples, the values of porosity with respect to the sample volume reached were as follows: D1—0.74%, D2—1.09%, and D3—1.17%.

From the analysis of the porosity of the samples for the tensile test, the authors found that when combining the layers’ deposition angles at values of 0°, 45°, 90°, and 135° in one sample (Type A), the defects were distributed over the entire sample volume. In addition, the authors could also observe a higher number of defects in the area where sample narrowed. On the other hand, in the case of the type B sample, defects were caused by an uneven fiber distribution, and in the type C sample, defects were observed at 90° bending points of the fiber. In addition, if compared to the type D sample without carbon fiber reinforcement, the authors could confirm and, thus, state that by adding reinforcing fibers to the base material, the porosity of the samples increased, and this is something that has also been proven in studies such as [47,48], where the authors also investigated fiber-reinforced composite materials. Thus, after this conclusion, it is certain and verified for the authors of this paper that the reinforcement in the base material causes the formation of pores and has an effect on the interlaminar strength.

On the other side of things, according to Handwerker [49], carbon-fiber-reinforced parts have or may have better properties than parts made of aluminum. However, the main disadvantage in the production of parts from reinforced composites produced by additive technology is their usually weak interlaminar strength and high porosity. In addition, in the case of the type B sample, the authors also observed a gap in the volume of the part created during the deposition of the fiber; however, it is necessary to state that due to the fiber diameter, the software did not allow the fiber to be evenly distributed. One of the reasons for this effect was the shape of the manufactured part, given that the geometry and corner radii on the parts are often limiting conditions for the deposition of the continuous filament in the matrix. This, in consequence, often leads to the formation of defects in components of more complex shapes, as also stated in [50,51].

### 3.2. Tensile Test

The tensile test was performed according to the ASTM D638 standard. The device on which the test was performed was a universal testing machine, i.e., the LaborTech LabTest 5.250 SP1. The speed of movement of the crossbar during the test was set at 5 mm.min^−1^.

**Figure 11:**
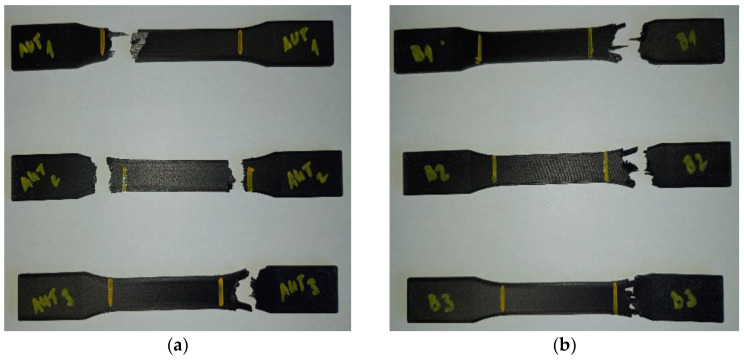
A take on the ruptured samples: (**a**) type A sample; (**b**) type B sample.

Figure 11 shows the individual sample types A and B after the tests. 

**Figure 12:**
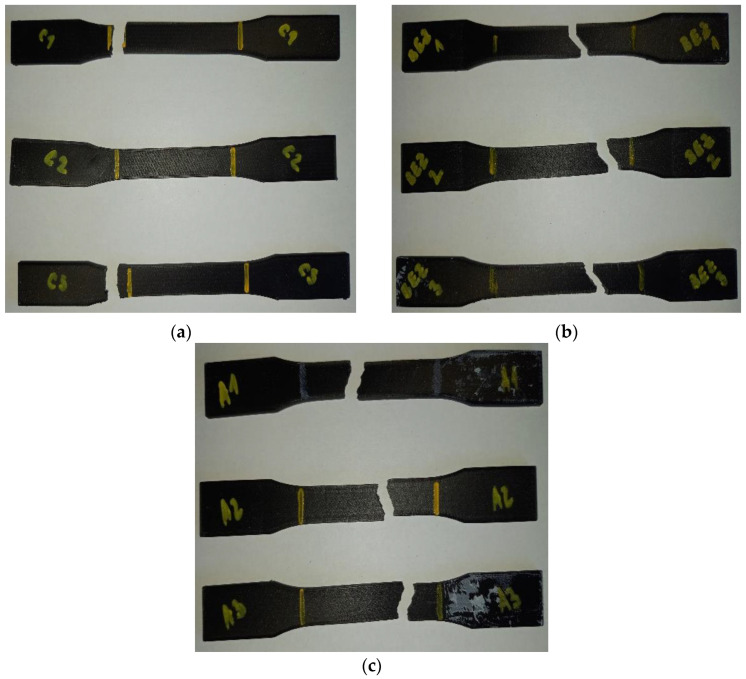
A take on the ruptured samples: (**a**) type C sample; (**b**) type D sample; (**c**) type E sample.

Figure 12 shows the individual sample types C, D, and E after the tests. 

As it can be appreciated in the whole of Figure 11 and in Figure 12a, the samples did not usually break in the narrowest part of the sample between the two yellow lines. This phenomenon was caused by the placement of the filament in the matrix during or when executing the calculations in the Eiger software. More specifically, this was caused when setting the printing parameters. When calculating the paths, the software is not always able to place fiber in all spots, due to the same shape of some parts, and then an uneven distribution of the fibers can, in consequence, take place. This issue may lead to having places without reinforcement in the part, which, of course, results in the weakening of these areas or places and, thus, in the subsequent failure of the sample.

As for Figure 12b,c, the type D and E samples broke where they were expected to break. The slight offset from the center of the part could have been caused by various factors, e.g., layer thickness, melting temperature, printing speed, and infill geometry. Another factor is the same fact that the Onyx material is a micro-carbon-fiber-filled nylon.

**Figure 13:**
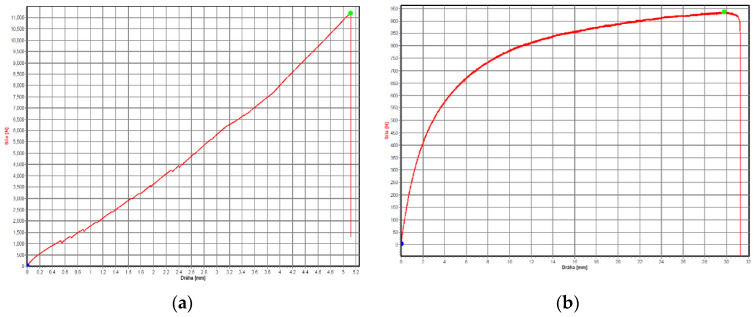
A general view of the tensile diagrams for samples: (**a**) type B sample; (**b**) type D.

On the other hand, Figure 13 shows examples of tensile diagrams for sample types B and D. 

Table 1 shows the measured values of the tensile strength Rm (MPa) obtained in the static tensile test for individual sample types. 

**Figure 14:**
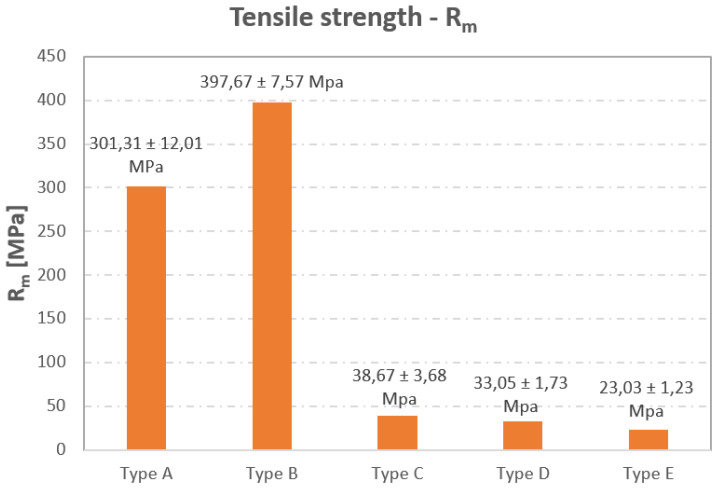
Resulting values of the tensile strength Rm (MPa) obtained in the static tensile test.

A summary of the results of the measured values is shown in Figure 14.

In Figure 14, it is possible to see that there is an increase in the tensile strength R_m_ of the type B sample; this value is approximately 12 times higher if compared to the Type D sample without reinforcement. Similar results have also been confirmed by other similar studies (see [52,53,54,55,56,57]). In the production of the type A sample, the authors used fiber deposition angles with values of 0°, 45°, 90°, and 135°, and, thus, each subsequent layer had a different fiber orientation. In this sense, the authors assumed that this sample would have the best tensile strength results; however, this did not prove to be true. This lower strength of the samples may be explained by the limitations that often occur during fiber deposition; for example, the fiber is either unevenly distributed in the volume of the matrix, or defects occur at the 90° bending point of the fiber, which, as mentioned before, leads to a higher number of pores in the composite. Other factors that affect the strength are also possible; for example: the layer thickness, the amount of fiber in the matrix, and also the fiber deposition itself [58,59]. In the case of the type C sample, the strength was also low. This was caused by an inappropriate orientation of the fiber with respect to the direction of the load, i.e., the fiber was oriented perpendicular to its direction, which usually leads to inferior strength values. Therefore, when designing composites, it is necessary to determine the direction of the load on the part as a whole; thus, based on this, it is then possible to define the correct deposition direction of the reinforcing fibers. 

As it is already known, by changing the orientation of the fibers, it is possible to change the mechanical properties of the components. This is even possible without changing the volume fraction of the fiber in the composite [60]. Composites where the fiber is oriented perpendicular to the direction of the load have the lowest strength, and this is related to the surface roughness of the part [61]. In the case of the type E sample (Infill 37%), this showed a decrease in strength compared to the type D sample (Solid Fill 100%). This was due to the lower volume of material, i.e., the filling of the sample, which was produced by the triangular mesh strategy with a filling of 37%.

### 3.3. Charpy Impact Test

The test was performed according to the ASTM E23 standard. The device on which the test was performed is an instrumented pendulum impact hammer of the type LaborTech CHK-300. Table 2 shows the measured values of the KV [J] work required for the individual sample types to break. 

**Figure 15:**
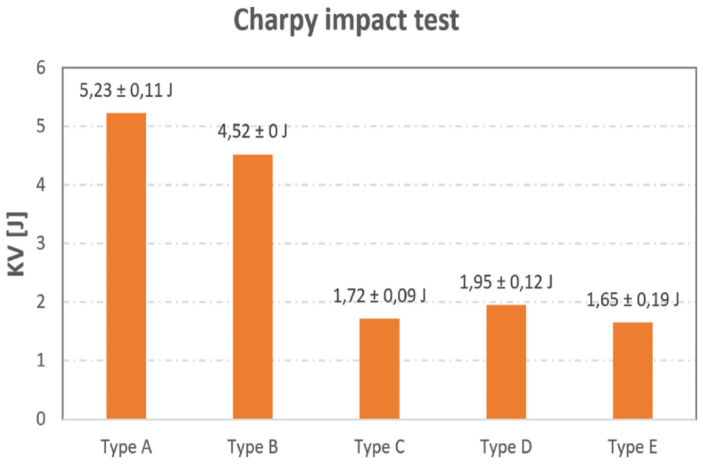
Resulting work values from the Charpy impact test.

The above-mentioned ASTM E23 standards define standard test methods for testing metal materials by impact using the Charpy impact test. During these tests, among other parameters, the amount of energy absorbed by a material during fracture is determined. Despite the fact that this standard is used for metallic materials, the authors decided to implement it for the case of the carbon-fiber-reinforced composite presented in this paper. It was the intention of the authors to monitor whether the fibers and their orientation affected the amount of work required to break the sample. From the results in Figure 15, it is clear that the fiber reinforcement did affect the work required to break the sample. In the case of the type A sample, a work of 5.23 J was required to break the specimen; this represented an increase of approximately 2.7 times in terms of the work required for the same purposes if compared to the Type D non-fiber sample.

**Figure 16:**
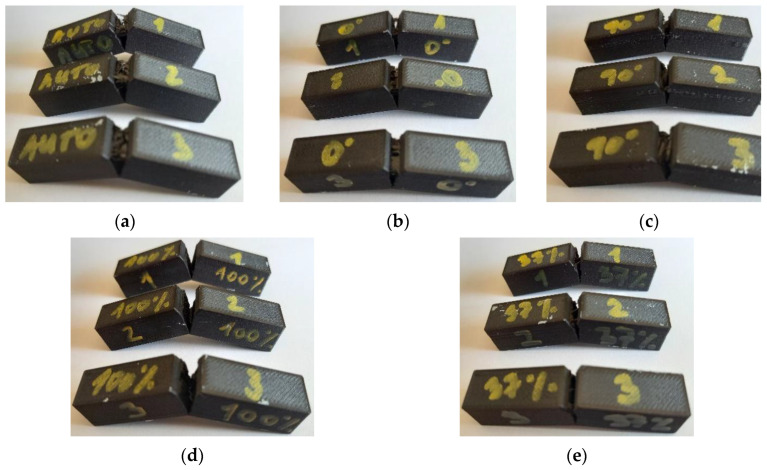
A general take on the tested samples: (**a**) Type A sample; (**b**) Type B sample; (**c**) Type C sample; (**d**) Type D sample; (**e**) Type E sample.

The Type B sample also saw approximately a 2.3 times increase in the work required to break. As in the tensile test, the authors can state and conclude that the fiber-reinforcement and their orientation did indeed affect the mechanical properties of the composite. On the other hand, in the case of the type C sample, a decrease of 0.23 J in terms of work was observed. This, like in the case of the tensile test specimens, was due to the unsuitable orientation of the fibers with respect to the direction of the applied force. This, in addition, may also be related to the adhesive characteristics of the fiber and the base material in the production of the samples themselves, which may have led and may indeed lead to delamination as also covered in [62]. Similarly, the decrease in work in the case of the type E sample (Infill 37%) was due to the different fill density compared to the type D sample (Solid Fill 100%). Figure 16 offers a general take on the tested samples of individual types A–E. 

The higher value of impact energy for the case of sample A can be explained by the fact that layers with different fiber orientations were placed on top of each other. In consequence, each of these layers became essentially stressed in a different direction during impact, which provided a certain advantage in this sense. Overall, this setup or configuration of all layers required higher energy to break the sample.

## 4. Conclusions

The aim of the research presented in this paper was to investigate the improvement of mechanical properties of composite materials with a controlled placement and/or orientation of the reinforcing element (carbon fiber). Reaching and verifying this goal also implied comparing these properties with those of unreinforced materials. In this sense, the achieved results and conclusions of this research can be summarized in the following three areas: 

(1) Porosity of reinforced composite material. In this case, the addition of carbon fiber to the base material was proven to increase the volume of defects in the sample. This came as a result of a porosity increase, which affected the same adhesive capacity of the fiber and the base material and had the potential to lead and indeed led to delamination of the individual layers. All of these facts are more evident and prone to occur in the case of more complex shape components. 

(2) As for the mechanical properties—tensile tests, the authors recorded an increase in the tensile strength Rm of up to 12 times higher compared to the sample without reinforcement. In this case, although the combination of several fiber orientations in one sample showed an increase in strength, these results did not achieve the highest strength among the tested samples, which was due to an improper placement of the fiber or their orientation, as it was discussed in detail in the respective section. This fact was exhibited by a sample with a fiber orientation at an angle of 0° (parallel to the direction of the load), i.e., a lower volume of material in the sample causes a decrease in strength, and this was also verified. 

The third results area referred to (3) mechanical properties—Charpy impact test. In this case, the fiber reinforcement was shown to affect the magnitude of work required to break the specimens. As in the tensile test, an increase in work of up to 2.7 times higher was recorded. An improper placement of the fiber in terms of its orientation was proven to reduce the strength, which was evident in the Type C sample, where the fibers were oriented at an angle of 0°, perpendicular to the direction of the force. In addition, it was also proven that, as with the tensile tests, a lower volume of material in the sample also led to a decrease in strength. 

The results of this paper overall indicate a clear need of further addressing the improvement of mechanical properties of composite materials. It was proven in this research that by controlling the orientation of the reinforcing elements (in our case, carbon fibers), it was also possible to control the properties of the end products, which is especially important during the analysis of the loading forces that act on the part or specimen under study. This fact is even more relevant and evident in the case of parts of complex shapes. It was also demonstrated that although the porosity of the part increases by inserting fibers into the base material, a better strength is still achieved. Even when this fact may be seen as a paradox from a classic metal-based point of view, i.e., porosity is never good when referring to metals, in the case of plastics, it all proved to be a benefit if the proper fiber orientation is taken into account as it was investigated and presented in this paper. This is a finding that has received poor attention and has been, up until this point, poorly explored in the analyzed literature. At the same time, it was also verified that there are other restrictions on the production of composite parts, which mainly relate to a reduced interlaminar strength and susceptibility to delamination. These effects were investigated, and it was concluded that these can be controlled by correctly combining and setting print parameters and fiber orientation. 

Through the research, the authors could also further explore and prove the advantages of using the Metrotom computed tomography for the purposes of monitoring the exact placement of the fibers in the material. It became evident that, based on the evaluation of the distribution of carbon fibers in the material, it is possible to analyze errors during the printing of the composite, which usually end up translating into material failure, as it was shown in Figure 11, where the samples were broken in the exact places where printing errors had been detected.

The authors’ future research will continue to focus on the application of fibers to complex-shaped prototypes and components. The authors would also like to investigate the use of other fillers coming from waste (see, for example, an interesting take presented by [63]), making clear comparisons to the ones investigated within this paper and, thus, exploring more about the capabilities and drawbacks of polymeric composite materials. Further research could also incorporate other highly precise means such as the SEM analysis to complement the results obtained by computed tomography as well as to further study the differences, advantages, and disadvantages between both approaches in terms of unraveling the mechanism of fracture after performing the mechanical tests.

## Figures and Tables

**Table 1:** Strength values Rm for the individual sample types.

Sample	Number			Average R_m_ Value (MPa)
	1	2	3	
Type A	287.91	311.12	304.89	301.31 ± 12.01 MPa
Type B	400.85	389.12	403.15	397.67 ± 7.57 MPa
Type C	42.86	35.95	37.19	38.67 ± 3.68 MPa
Type D	33.89	33.76	31.49	33.05 ± 1.73 MPa
Type E	21.88	24.32	22.88	23.03 ± 1.23 MPa

**Table 2:** Values of work in KV [J] required to break the test specimen.

Sample	Number			Average Work Values KV [J]
	1	2	3	
Type A	5.13	5.34	5.23	5.23 ± 0.11 J
Type B	4.52	4.52	4.52	4.52 ± 0 J
Type C	1.82	1.67	1.67	1.72 ± 0.09 J
Type D	1.82	2.05	1.97	1.95 ± 0.12 J
Type E	1.44	1.71	1.80	1.65 ± 0.19 J

## Data Availability

Not applicable.
